# Town and Country

**Published:** 1893-04

**Authors:** Kirke White


### TOWN AND COUNTRY.

II.

It is amusing, sometimes, about seven o'clock on a summer morning to see, perhaps a couple of old red-cowled men, shoe makers, perhaps, or small shopkeepers, taking a stroll with their hands in their pockets under their aprons, about three hundred yards out of town, where, amidst the villas of the suburbs, there may flourish a mere remnant of a field covered with growing wheat; they inspect this with a calculating air, and have their own unprofessional remarks on its appearance. And then they come back to town, and talk for a week about the crops.

Or on a summer Sunday evening, when all the fashionable, and even what is called the respectable world, keeps haughtily within doors, how delightful it is to see the honest mechanic taking a stroll in some of the highways or byways a little way out of town, along with his wife and perhaps one or two of his children! His clothes are decent, though perhaps deficient in expression and still marked with the creases incurred in their ordinary day sinecurism.

He probably carries his youngest child in his arms, while the wife sails on, broad and large in a red shawl, in front, attended by a few walking youngsters, who are ever and anon asking her questions about some rural object that strikes their eyes for the first time.

It is about six o'clock, and though the house is deserted and locked up, the kettle has been left very near the smothered fire so as to be ready when they return, for the infusion of the tea which is destined to conclude the day's humble and well earned enjoyments. As he moves along, with his arms clasped close around his little one, and the back of his coat swinging loose and free of his back, he prattles with his straggling family group about all the cows, and the horses and the farmyards they come in sight of; and ever and anon he takes an interested and knowing look at the wheat fields, as if he saw in them the shadow of his coming loaf, and wondered what flour would cost per sack next year. If the weather be clear, with the sun shining over head, then he rejoices for two reasons,—it is pleasant for a walk and it promises to "do good to the country." If a shower comes on, it discomposes him and his family not a little and drives them, a little draggled perhaps, into the next public house; yet he suffers all with a good grace and a resigned heart, "it may, perhaps, be of good to the country." If, in passing along, he sees a boy intruding upon a piece of grain, for the purpose of plucking and bruising it, he cries to him in an authoritative tone of voice, to come out of "the victual;" this last phrase being the one which he is disposed to apply to grain when he wants to treat it with more than usual respect. Whenever the group comes to a place where the enclosing fences are somewhat lofty, then has he to lift up his children, one by one, that they may look over and see what they can see.

The wife occasionally asks questions about the neighboring seats, and, in talking of these unusual matters, a style of speech steals unconsciously upon the worthy couple, which is considerably different in tone and language from what they use at home on the week-days.

They feel somewhat like civil strangers in a higher rank of life, explaining things to each other in an urbane and genteel kind of manner, and it is not until they reach home and recommence household realities, that they become exactly as familiar as they usually are with each other.

It is the most ridiculous thing in the world to institute invidious comparisons between the country and the town, or to say that the balance of advantage lies on either side. Cowper's celebrated line "God made the country, but man made the town," is a fallacy. Much of both the country and the town is the creation of the Almighty and much of both is the creation, in a certain sense, of man. The fields are rendered by man very different from what they were originally; and though his handiwork is more observable in the city, still there is only a difference in degree. The institutions of social life prevail in both town and country; and though there is perhaps more sophistication in the former, still that, too, is only a difference in degree.

If we concede that social life was intended as the proper condition of man, we must allow that the clustering of certain of the race in cities must have been expressly contemplated from the first as the dispersion of others over the face of nature, for the existence of masses of population is a necessary consequence of social life.

In this, as in everything else, man has his choice. If he prefers the air and sights of the sweet-breathed country to the conventional conveniences of a city, he is right for himself and for his kind.

If he prefers these conventional conveniences, at the expense of some of the said air and lights, then he is right too. For by either way the general good is advanced.

In short, we would like to see all sorts of people removed above inconsiderate prejudices respecting the lot and choice of their neighbors. Even to WONDER how another man lives, wanting the things which YOU appreciate in your own destiny, shows an absence of proper reflection and would be as well avoided.

No man can know what happiness there is in the condition of his fellowmen, unless he put himself into the same situation. Then he is apt to find that, in the sphere and caste where he formerly thought there was nothing but unmingled misery, there exist many unseen comforts and blessings which redeem its outward aspects.

KIRKE WHITE.

